# Relationship Between Knowledge and Types of Attitudes Towards People Living with Dementia

**DOI:** 10.3390/ijerph17113777

**Published:** 2020-05-26

**Authors:** Chia-Yu Chang, Hui-Chuan Hsu

**Affiliations:** 1 Department of Healthcare Administration, Asia University, Taichung 41354, Taiwan; chiayuchang2@gmail.com; 2 South District Public Health Center, Taichung City Government, Taichung 402332, Taiwan; 3 School of Public Health, Research Center of Health Equity, College of Public Health, Taipei Medical University, Taipei 11031, Taiwan

**Keywords:** attitudes, dementia, dementia-friendly community, people living with dementia, text mining, stigma

## Abstract

The purpose of this study was to examine the relationship between knowledge and attitudes towards dementia among adults. A cross-sectional online survey with purposive sampling was conducted in four district health centers in Taichung, Taiwan, in 2018. Knowledge was measured by the Taiwanese version of the Dementia Knowledge Assessment Scale. Attitudes towards people with dementia were measured by four kinds of feelings: discomfort, shame, avoidance, and fear. In total, 347 persons completed the questionnaire. Knowledge of dementia was modest. Higher education, a care-related background, chronic health conditions, experience caring for people with dementia, and knowledge of family history were related to higher knowledge of dementia. Attitudes towards people with dementia were grouped into four clusters: uncomfortable (22.2%), ashamed (2.6%), unfriendly (22.5%), and non-negative (52.7%). Knowledge of dementia was significantly related to the ashamed cluster but not to the other clusters. Lower education, a lack of caring experience, and a lack of knowledge of family history were related to uncomfortable feelings, and poorer financial satisfaction was related to uncomfortable, afraid, and avoidant feelings. The open-question expression of feelings towards dementia was more likely to be negative (55.3%). The public should be educated on knowledge of and friendly attitudes towards dementia.

## 1. Introduction

The prevalence of dementia has increased dramatically with the global population aging. In 2017, nearly 50 million people worldwide had dementia, and this number is expected to grow to 131.5 million people by 2050 [1]. In Taiwan, the prevalence of mild cognitive impairment in the elderly was 18.76% in 2013, and the dementia prevalence was 8.04%; the rate doubled for every 5-year increase after age 75 [2]. However, people are still less familiar with dementia than with other chronic diseases among older adults. Not only do lay people lack knowledge about dementia, but professionals also have misunderstandings about the differences between normal aging and dementia [3]. Additionally, stigma may prevent early diagnosis, timely treatment, and appropriate quality of care for people with dementia [4,5,6,7]. Promoting awareness and removing the stigma of dementia are the priorities for promoting a dementia-friendly community. Alzheimer’s Disease International (ADI) [8] defines a dementia-friendly community as “a place or culture in which people with dementia and their carers are empowered, supported, and included in society, understand their rights and recognize their full potential.” ADI suggests four elements as the cornerstone of a dementia-friendly community: people (involvement of people living with dementia, or PWDs), communities (the social environment and the physical environment), organizations (dementia-friendly organizations, access to appropriate health care), and partnerships. However, research on dementia friendliness has just begun in recent years, and the relationship between knowledge of and attitudes towards dementia remains unclear.

### 1.1. Knowledge of Dementia

Many studies have been conducted on knowledge of dementia. A systematic review has shown that the majority of people have only modest to fair knowledge about dementia, and a common misconception is thinking that dementia is part of the normal aging process [6,9]. Better knowledge of dementia was found to be related to training or background, higher social class, higher education, being younger, residing in an urban area, and experience with dementia contact or care [10,11,12,13].

### 1.2. Attitudes Towards Dementia

A negative attitude towards dementia is related to ageism and the fear of psychiatric syndromes. When such a negative attitude becomes severe and widespread among the population, stigma related to dementia may prevent understanding and compassion towards people living with dementia. Stigma is a social experience that refers to the perception of others that an individual is deviant from the norm [14]. There are three kinds of stigma: public stigma (the general public carries a negative belief, such as stigma towards dementia), self-stigma (felt or internalized negative belief, such as PWDs’ perceptions), and spillover stigma (negative experience due to social proximity, such as caregivers of PWDs) [14].

The concept of dementia friendliness has recently emerged, and measures of dementia friendliness have been developed. Griffiths et al. defined the domains of attitudes based on factor analysis, including perceptions of dementia, empathy towards PWDs, and personal sacrifice in caring for a PWD [15]. Phillipson et al. explored attitudes in terms of personal avoidance, fear of labeling and fear of discrimination, and person centeredness [16]. Piver et al. investigated the stigma of Alzheimer’s disease using five domains of 10 items: reluctance towards the disease, emotional impact, fear of exclusion, courtesy stigma, and loss of family support [17]. Rosato et al. used 13 words relating to positive/negative feelings towards dementia and used latent class analysis to identify three groups of attitudes: mixed antipathy/empathy, high antipathy, and moderate antipathy [12]. The findings indicate that attitudes towards dementia may be mixed and involve multiple feelings. People who report having a good relationship with a PWD may still feel ashamed if they have a family member with dementia [18]. General factors related to attitudes towards dementia are sex, age, knowing someone with dementia, caring experience, residence area, and neighborhood trust or support [13,16,17,19].

It remains unclear whether health care professionals have a friendlier attitude towards PWDs than lay people do. Earlier research reported that health professionals may perpetuate stigma [17,20], and especially when health professionals have the power to diagnose and label the disease, the stigma of dementia is likely to occur [14]. A study indicated that general practitioners believe that they have the main responsibility for the diagnosis of dementia, and late diagnosis may be related to the stigma of making the diagnosis [21]. Professional stigma may cause failure to provide early diagnosis, and this stigma may be mixed with ethnic discrimination [6]. Social exclusion or stigma by professionals may be more severe in minority groups of PWDs. However, recent studies have shown that health professionals have a friendlier attitude toward PWDs than volunteers do [18,20]. The awareness of dementia-friendliness may have been developing in professionals.

The neighborhood environment is also related to attitudes towards dementia, and people in less-developed areas may be more supportive. Rural areas may not have sufficient access to medical care for dementia, but the positive aspect of rural areas is the pleasant, natural environment with supportive ambiance [22]. One study reported that although aboriginal PWDs in Taiwan had lower awareness of dementia, they viewed dementia as a condition but not a disease, and the family-like ambience in the community allowed them to continue their daily lives as usual [23]. A dementia-friendly neighborhood may have protective effects for cognitive decline and act as a buffer between low education and cognitive impairment [19]. However, it is also possible that more severely cognitively impaired individuals do not receive enough social support. This phenomenon requires further research. Although a dementia-friendly neighborhood is important, the barrier of a lack of formal resources may not be sustainable [24]. The general population’s anxiety about future dementia care is related to demographic characteristics and a lack of social capital; access to general practitioners is not significant [25]. This also implies that informal support (from family and neighbors) is the core of dementia friendliness because dementia care occurs on a daily basis.

### 1.3. Knowledge and Attitudes

A systematic review found that a stigmatizing attitude is usually related to limited disease knowledge, little contact experience, the male sex, and younger age in the context of ethnicity and culture [20]. However, the relationship between knowledge and attitudes is unclear. A study in Ireland found that greater knowledge about dementia did not ensure a friendlier attitude towards people living with dementia, and there may even be a negative relationship between awareness and friendliness [12]. In the study by Ebert, Kulibert, and McFadden [26], biomedical knowledge of dementia was not significantly related to fear of dementia, but social comfort was negatively related to personal fear of dementia. This finding implies that knowledge of dementia is not necessarily essential to develop a dementia-friendly community but sharing experience in the community is helpful to increase friendliness.

The relationship between low knowledge and a friendly attitude has also been found in different cultures. Previous research has indicated that people who are older and less educated are more likely to have stigma towards people with dementia [27]. Another study in Pakistan showed that PWDs felt they were treated well by their family and neighbors, and the stigma was low. Dementia symptoms may interfere with religious practices, but family caregivers just cope and trust God’s will [28].

Although awareness of and friendliness towards dementia have been examined in recent years, there are some research gaps. First, the domains and the measures of attitudes towards dementia or stigma are still under development. Attitude is often measured by a single score as positive or negative, but research on different domains or feelings of stigma is limited. Second, many quantitative or qualitative studies have been conducted, but a mixed methodology has rarely been applied. Third, the relationship between knowledge of and attitudes towards dementia has not yet been confirmed.

Thus, the purpose of this study was to explore knowledge of and attitudes towards dementia among the general population. In this study, we examined knowledge of and attitudes towards dementia among adults in the community in Taichung, Taiwan, using a survey. The structural questions analyzed the relationship between knowledge and attitudes towards dementia, and the answers to the open-ended question about attitudes towards dementia were analyzed by text mining. The findings are expected to provide implications to promote awareness of and friendliness towards dementia.

## 2. Materials and Methods

### 2.1. Data and Sample

A cross-sectional survey was conducted to collect data on knowledge of and attitudes towards people living with dementia. The participants were selected by purposive sampling from 4 public health centers in Taichung, Taiwan. We invited the people and their company members who came to the 4 public health centers for health check-ups, health education, health promotion, activities, or screening activities to be the participants. The inclusion criteria were those who were aged 20 years or older; able to communicate and read; and able to use computers, tablets, or mobile phones. The participants were invited at the health care centers, and the study was introduced by the researcher. All the invited participants finished the questionnaires, and no one rejected to participate in the survey. Once participants agreed to participate in the study, the interviews were conducted. One computer and one tablet were provided at the public health centers. The participants completed the questionnaire independently. Assistance in using the Internet was provided if necessary. In total, 347 persons completed the questionnaire from August 2018 to December 2018.

### 2.2. Measures

#### 2.2.1. Taiwanese Version of the Dementia Knowledge Assessment Scale (T-DKAS)

Knowledge of dementia was translated from the Dementia Knowledge Assessment Scale (DKAS) [29,30]. The original DKAS consists of multiple domains of knowledge about dementia, including 25 items and 4 subscales: causes and characteristics (7 items), communication and behavior (6 items), care considerations (6 items), and risk factors and health promotion (6 items). We obtained approval for the translation of DKAS first and then started the translation process. The original DKAS was translated to Chinese by a public health Ph.D. of the University of Cambridge, who was a Chinese native speaker, and then a dementia care specialty physician conducted the back translation of the Chinese version of the scale to English. Three registered nurses with dementia care experience were also invited to provide suggestions for the translated scale. Options for each item were comprised of wrong, possibly wrong, possibly correct, correct, and not known. The items were recorded as incorrect/correct (score 0/1), and a total score was calculated ranging from 0 to 25.

The Taiwanese version of the DKAS (T-DKAS) was pretested on 150 adults of different age groups and backgrounds. A test-retest was also conducted on 35 persons. The Cronbach’s α was 0.782, and the test-retest reliability estimated by Pearson’s correlation coefficient was acceptable (*r* = 0.847, *p* < 0.001), with agreement of 64.91–94.74%.

#### 2.2.2. Attitudes Towards People Living with Dementia

Attitudes towards people living with dementia included four closed questions and one open question. The items of the 4 closed questions were as follows: shame (do you feel shame if your family members have dementia), fear (are you afraid of spending time with family/friends/neighbors living with dementia), avoidance (do you avoid being with family/friends/neighbors living with dementia), and discomfort (do you feel comfortable with family/friends/neighbors living with dementia). Each item was coded as yes/no. The items were reviewed by three dementia care and health behavior experts to assure face validity. The participants were also asked their feelings about dementia through an open question with 3 words or phrases.

#### 2.2.3. Control Variables

The control variables included demographics and health status: age, sex (male/female), education (under high school or college and above), marital status (having spouse or not), occupation (no job, medical/social care-related occupation, other occupation), living arrangement (living alone or living with others), subjective financial satisfaction (poor/good), self-rated health (poor, fair, good), chronic health conditions (yes/no), dementia family history (yes/no), and experience in taking care of a person living with dementia (yes/no).

### 2.3. Ethical Considerations

All participants participated in the survey after they understood the content and agreed to participate. This study was approved by the Medical Research Ethics Committee of Asia University (No. 1061229) before the study was conducted.

### 2.4. Analysis

The analysis methods included descriptive analysis, linear regression, cluster analysis, and multinomial logistic regression. Factors related to knowledge of dementia were analyzed by linear regression. Attitudes towards dementia were first explored by hierarchical cluster analysis and then grouped by k-means cluster analysis. Then, multinomial logistic regression analysis was conducted to examine the related factors with clusters of attitudes towards dementia. The analysis was conducted with PASW (SPSS) 18.0 software (SPSS, Chicago, IL, USA).

The answers to the open question of attitudes towards dementia were recorded in a text file. The words were first classified as positive, neutral, or negative by the authors. Negative words were defined as stereotypes of dementia as unfriendly according to the 2019 World Alzheimer’s Report: Attitudes to Dementia [3]. The symptoms of dementia were classified as neutral. Next, the text data were analyzed by text mining using Python software (version 3.7.6) (Python, Wilmington, DE, USA). Descriptive analysis and word clouds were applied.

## 3. Results

The characteristics of the sample are shown in Table 1. The mean age of the sample was 44.7 years, and 13.3% were 60 years old or older. Females (53.6%) were slightly more prevalent than males (46.4%). Approximately one-third (32.3%) had experience caring for PWDs. The average score of dementia knowledge was 15.7 (the total score ranged from 0 to 25). Regarding the four types of attitudes towards dementia, 10.1% felt ashamed, 79.8% were afraid to spend time with PWDs, 82.1% avoided being with PWDs, and 55.9% felt uncomfortable with PWDs.

Table 2 examines the factors related to knowledge of dementia by linear regression, with explained variance = 29.4%. Participants who had higher education and medical/social care-related occupations, no chronic conditions, and experience in caring for PWDs had more knowledge of dementia. Those who did not know whether they had a family history of dementia also had lower knowledge of dementia.

The original distribution of the four items of attitudes towards dementia is shown in the Supplementary Tables S1 and S2. There were 12 kinds of attitudes of 16 possible combinations. Most people (50.4%) were classified as no discomfort, no avoidance, no shame, and no fear. We first used hierarchical cluster analysis to explore the possible clusters and classify them into four clusters. Then, k-means cluster analysis was conducted to obtain four clusters of participants, as shown in Table 3. The four clusters (and the percentage of the participants) were called uncomfortable only (feeling uncomfortable with PWDs who are family/friends/neighbors) (22.19%), ashamed (feeling ashamed if family members have dementia) (2.6%), unfriendly (feeling uncomfortable, afraid, and avoid being with PWDs) (22.5%), and non-negative (no feelings of shame, fear, avoidance, and discomfort) (52.7%). It means that almost half (47.3%) of the participants had negative feelings to PWDs, and the unfriendly group occupied more than one fifth.

Table 4 shows the factors related to attitudes towards dementia by multinomial logistic regression models; the reference group of attitudes was the non-negative group. Compared with the non-negative cluster, participants in the uncomfortable only cluster (cluster 1) were more likely to be less educated (OR = 2.213, *p* < 0.05) and have a family history of dementia (OR = 3.604, *p* < 0.01). Compared with the non-negative attitude cluster, participants in the ashamed cluster (cluster 2) were more likely to be younger (OR = 0.254, *p* < 0.05), have chronic health conditions (OR = 0.045, *p* < 0.05), have experience caring for PWDs (OR = 0.041, *p* < 0.05), and have less knowledge of dementia (OR = 0.612, *p* < 0.01). All the participants in this cluster had a family history of dementia; thus, this variable was not included in the model. Participants in the unfriendly cluster (cluster 3) were more likely to have poor financial satisfaction than those in the non-negative cluster (OR = 2.469, *p* < 0.01).

The participants were also asked by an open question about their feelings towards dementia. There were 347 persons who responded to this question and provided 1041 words about dementia. The characteristics of the words were classified by the authors. More than half (55.3%) of the words were negative, such as helpless, pitiful, depressed, terrifying, and horrifying. Only 17.8% of the words were positive, such as caregiving, empathy, and cute. In addition, 23.1% of the words were neutral and were mostly related to symptoms such as forgetting things, repetitive behavior, and missing.

**Figure 1:**
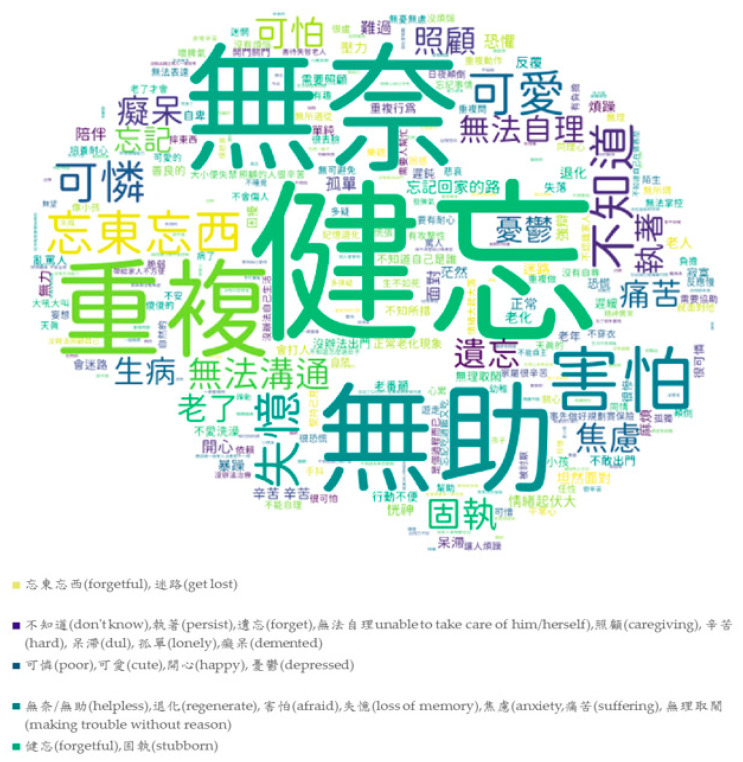
Word clouds of feelings toward dementia. Note: Translation of frequent Chinese characters: 健忘/忘東忘西/忘記/失憶/遺忘(forgetful, forget, loss of memory), 害怕(afraid)/恐懼(fear), 無助/無奈(helpless), 可憐(pitiful), 照顧(caregiving), 可怕(terrifying), 憂鬱(depression), 焦慮(anxiety), 重複(repeatedly), 迷路/忘記回家的路(get lost), 辛苦(hard), 無法溝通(incommunicable), 固執(stubborn), 退化(regenerate), 麻煩(trouble), 痛苦(suffering), 不知道(don’t know), 正常老化/老了(normal aging), 可愛(cute), 開心(happy), 無法自理(unable to take care of him/herself), 孤單(lonely).

Figure 1 shows the word clouds of feelings towards dementia by an open question that shows the most frequent words from the recorded texts. The translation of the Chinese characters is shown in the note for Figure 1. We translated the words/phrases into English in the note of Figure 1, and the color legends also show the translation of the words/phrases with similar colors. The colors of the words were assigned by the software randomly, and the size of the words represents the frequency. The bigger words mean the words were more frequently mentioned in the responses. The most frequent words were about symptoms of dementia (forgetful, forget, loss of memory, repeatedly, unable to communicate, stubborn, and get lost), thoughts about caregiving (caregiving, unable to take care of him/her, need assistance, accompany, hard), negative feelings towards dementia or PWDs (such as afraid/fear, terrifying, helpless, pitiful, helpless, like a child, sad, trouble, anxiety, suffering, lonely, and nothing you can do), and positive feelings (such as cute and happy).

## 4. Discussion

This study conducted a survey in an adult sample in health centers in Taichung, Taiwan, to examine the participants’ knowledge and attitudes towards dementia. The level of knowledge was modest. Four attitudes (shame, fear, avoidance, and discomfort) were examined, and the participants were clustered according to the attitudes into four groups: uncomfortable (22.2%), ashamed (2.6%), unfriendly (22.5%), and non-negative (52.7%). More than half of the participants did not hold a negative attitude towards PWDs. Knowledge of dementia was only significantly related to the ashamed cluster; having higher knowledge was related to a lower likelihood of feeling ashamed. Negative expressions of feelings towards dementia based on the open responses constituted more than half of the words.

### 4.1. Knowledge of Dementia

The participants’ knowledge of dementia was modest, as found in another study [9]. The significant factors related to better knowledge of dementia included higher education, experience caring for PWDs and a family history, and related professional backgrounds, consistent with previous studies [10,11,12,13]. This implies that health literacy is related to training or education. It is suggested that health literacy about dementia should be included in general health education, or the education may be delivered to the public in various forms for the public, such as films, videos in social media, and popular songs for the public. Age was not significantly different from previous studies; a younger age likely indicates more education. Chronic diseases were found to be related to more knowledge of dementia. It is possible that people with chronic diseases are more aware of the risks of all chronic diseases, including dementia. In Taiwan, some chronic disease patients are included in a complete management program for their disease, such as diabetes and hypertension. They may receive more health education information than others.

### 4.2. Attitudes Towards Dementia

In this study, we used four variables to describe attitudes towards dementia and identified four types of attitudes. In past research, most studies used only positive–negative scores to describe attitudes towards dementia. The different domains of attitudes towards dementia were explored by Griffiths et al., Phillipson et al., Piver et al., and Rosato et al. [12,15,16,17]; some of the domains are not attitudes but are also mixed with actions. The measures of attitude towards dementia in our study were similar to those in previous studies, such as avoidance and shame [16] and avoidance and discomfort [15]. Our measures of attitudes towards dementia focused only on stigma. The strategy to define the typology of attitudes towards dementia in our study was similar to the study of Rosato et al. [12], which used latent class analysis to categorize participants into a mix of empathy and antipathy. Our study used four variables to describe the types of feelings towards dementia by cluster analysis, and we demonstrated more complicated feelings by identifying types as uncomfortable, ashamed, unfriendly, and non-negative.

Approximately half of the participants were classified as the non-negative group, which means that the public is overly discriminatory towards PWDs if these responses were honest. It is also possible that those who did not have experience in caring for PWDs or who did not have a family history did not know how they would feel in all circumstances. The unfriendly group, which had feelings of fear, avoidance, and discomfort, constituted 22.5%. The uncomfortable only group constituted 22.2%. Those who had poor financial status were more likely to be unfriendly, and those who were less educated were more likely to be uncomfortable only when controlling for other variables. One possible reason is that lower socioeconomic status is usually related to lower education and less health literacy, and misunderstandings of dementia cause unfriendly feelings. Another explanation is that lower socioeconomic status means less resources and ability to care for PWDs. If people do not know how to take care of PWDs and do not know where to get assistance, problems with caregiving will produce negative and unfriendly feelings. In addition, the uncomfortable only group was related to having a family history. Because the PWDs were their family, they were unlikely to show more severe stigma (fear and avoidance); they only felt uncomfortable. If they know how to get along with PWDs, they can show their empathy to PWDs.

Better knowledge was more likely to be related to a non-negative attitude than feelings of shame. The ashamed group of participants all had a family history of dementia, and they had lower knowledge about dementia. This means that the self-stigma of dementia would be an issue for the family caregivers of PWDs to search more information about dementia. This may cause reluctance to seek help or care for dementia. Knowledge was not significantly related to the other attitude groups. This finding implies that having more clinical knowledge about the disease does not necessarily produce a friendlier attitude. It seems that knowledge and attitudes are independent domains in certain social and cultural environments. Unless friendliness towards dementia is included in health education, knowledge about the disease itself may not improve friendliness towards dementia.

In this study, we also used text mining to express feelings about dementia by word clouds. Although the symptoms that described dementia constituted most of the impressions of dementia, the respondents often used negative words to describe their feelings towards dementia. Although negative words occurred more frequently than neutral and positive words, we did not see many discriminatory words in the word clouds. The expressions were more related to the feeling of having dementia or the caregiving burden of dementia. It seems that the participants also worried about care if they developed dementia. If options for dementia care are available and the knowledge and ability to care for PWDs and the quality of care improve, concerns and fears about morbidity or caregiving for PWDs may also be reduced.

### 4.3. Limitations

There are some limitations of this study. First, the measure of attitudes towards dementia focused on stigma; positive feelings and additional domains were not included. The measure of attitudes towards dementia may not be comprehensive. Second, a purposive sampling in several health centers in one city was conducted in this study. Only those who were able to communicate with and able to use devices with Internet were included. The results may not be generalizable to other populations, and the sample size was not large. Third, this study was cross-sectional; a causal relationship cannot be confirmed. Fourth, a more qualitative description of feelings towards dementia should be explored. The expression of three terms may not be sufficient to show deeper feelings towards dementia. In fact, qualitative in-depth interviews were also conducted on this sample. The triangulation of qualitative and quantitative analyses should be conducted in the future to provide more information about dementia friendliness. The qualitative expressions can be further analyzed by semantic analysis to identify different feelings towards dementia rather than simply classifying them as positive, neutral, and negative.

## 5. Conclusions

The level of knowledge about dementia is still modest. Fear and avoidance are common, and four types of attitudes towards dementia were identified. Although attitudes or stigma towards dementia are deeply rooted, it is possible to change these attitudes through interventions using education and more interactions with PWDs [31,32]. We suggest the promotion of more health education for the public about disease knowledge as well as dementia friendliness. Furthermore, dementia friendliness should be included in medical education for professionals and in mandatory education for all students and delivered as education messages through multiple media to the public.

## Figures and Tables

**Table 1:** Description of the sample.

Variables	Mean (SD) or %
Gender	
Male	46.4
Female	53.6
Age	44.7 (12.1)
20–29	12.4
30–39	25.4
40–49	23.6
50–59	25.4
Above 60	13.3
Marital status	
No spouse	44.4
Having spouse	55.6
Education	
Under high school	36.6
College and above	63.4
Occupation	
Non-related background	59.7
Medical- or social care-related	21.9
Not working	18.4
Living arrangement	
Living alone	9.2
Living with others	90.8
Subjective financial satisfaction	
Poor	42.1
Good	57.9
Self-rated health	
Poor	12.4
Fair	43.5
Good	44.1
Chronic health conditions	
No	71.8
Yes	28.2
Dementia family history	
No	77.5
Yes	12.1
Don’t know	10.4
Experience in caring for a PWD	
No	67.7
Yes	32.3
Dementia Knowledge Assessment Score (score 0–25)	15.7 (4.6)
Attitudes towards people living with dementia	
Shame	
No	89.9
Yes	10.1
Fear	
No	20.2
Yes	79.8
Avoidance	
No	17.9
Yes	82.1
Discomfort	
No	44.1
Yes	55.9

Note: *n* = 347.

**Table 2:** Factors related to knowledge about dementia by multivariate linear regression.

Variables	Βeta	Standard Error
Constant	18.683	0.941 ***
Age	−0.151	0.211
Gender (female)	0.687	0.524
Marital status (no spouse)	0.147	0.462
Education (high school or lower)	−1.942	0.486 ***
Occupation		
Medical- or social care-related	2.529	0.787 **
Not care-related	0.213	0.649
Financial satisfaction (poor)	0.240	0.458
Chronic health conditions (no)	−1.360	0.483 **
Experience in caring for a PWD (no)	−2.596	0.518 ***
Dementia family history		
Yes	−1.147	0.687
Don’t know	−1.719	0.720 *
*Explained variance R^2^*	0.294	

Note: *n* = 347. The reference groups were: gender (male), marital status (having spouse), education (college and above), occupation (not working), financial satisfaction (good), chronic health conditions (yes), experience in caring for a person living with dementia (yes), dementia family history (no). Age was an ordinal variable. The analysis was carried out by liner regression. * *p* < 0.05, ** *p* < 0.01, *** *p* < 0.001.

**Table 3:** Cluster analysis according to the attitudes towards dementia of the participants.

Variables	Cluster 1 Uncomfortable (*n* = 77, 22.2%)	Cluster 2 Ashamed (*n* = 9, 2.6%)	Cluster 3 Unfriendly (*n* = 78, 22.5%)	Cluster 4 Non-negative (*n* = 183, 52.7%)
Shame	0.17	1.00	0.17	0.00
Fear	0.00	0.00	0.79	0.04
Avoidance	0.00	0.11	0.78	0.00
Discomfort	1.00	0.00	0.97	0.00

Note: *n* = 347.

**Table 4:** Factors related to attitudes about dementia by multinomial logistic regression.

**Variables**	**Cluster 1: Uncomfortable only** **(Feeling Uncomfortable with PWD Family/Friends)**			**Cluster 2: Ashamed (Feeling Ashamed if Family Members Have Dementia)**			**Cluster 3: Unfriendly (Feeling Uncomfortable, Afraid, and Would Avoid getting Along with a PWD)**		
	Odds Ratio	95% CI of OR		Odds Ratio	95% CI of OR		Odds Ratio	95% CI of OR	
	OR	Lower	Upper	OR	Lower	Upper	OR	Lower	Upper
Age	0.943	0.708	1.257	0.254 *	0.065	0.995	1.077	0.813	1.426
Gender (female)	0.996	0.499	1.986	1.179	0.126	11.008	0.980	0.498	1.927
Marital status (no spouse)	0.615	0.330	1.149	0.113	0.010	1.306	0.782	0.429	1.427
Education (high school or lower)	2.213 *	1.156	4.235	0.305	0.026	3.521	1.295	0.684	2.452
Occupation									
Occupation (not care-related)	1.483	0.637	3.452	0.325	0.015	7.055	1.345	0.587	3.081
Occupation (medical/social care-related)	0.584	0.182	1.872	1.386	0.065	29.728	0.637	0.212	1.919
Financial satisfaction (poor)	0.894	0.478	1.671	1.607	0.341	7.572	2.469 **	1.355	4.499
Chronic health conditions (no)	1.235	0.630	2.419	0.045 *	0.004	0.575	1.010	0.529	1.928
Caring for PWD experience (no)	2.042	0.957	4.357	0.041 *	0.002	0.694	1.520	0.737	3.136
Dementia family history									
Dementia family history (yes)	3.604 **	1.485	8.745	-	-	-	0.934	0.320	2.727
Dementia family history (don’t know)	1.613	0.622	4.182	-	-	-	1.014	0.405	2.534
Knowledge of dementia score	0.994	0.925	1.069	0.612 **	0.424	0.883	0.971	0.905	1.043

Note: *n* = 347. The reference group: Attitude toward dementia (non-negative), gender (male), marital status (having spouse), education (college and above), occupation (not working), financial satisfaction (good), chronic health conditions (any), caring for PWD experience (yes), dementia family history (no). Age was an ordinal variable. The analysis was conducted by multinomial logistic regression. * *p* < 0.05, ** *p* < 0.01, *** *p* < 0.001.

## Supplementary Materials

The following are available online at https://www.mdpi.com/1660-4601/17/11/3777/s1, Table S1. Distribution of the attitude to dementia in two-by-two tables; Table S2. Distribution of all the combinations of attitudes to dementia.
