# Supplementary material for: Relationship Between Knowledge and Types of Attitudes Towards People Living with Dementia
Source: Int J Environ Res Public Health. 2020 May 26;17(11):3777. doi: 10.3390/ijerph17113777 (PMC7312095; doi:10.3390/ijerph17113777)
Supplement: Supplementary file 1 [file ijerph-17-03777-s001.pdf]

Table S1. Distribution of the attitude to dementia in two-by-two tables (persons and %).

		Shame		Fear		Avoidance	
		No	Yes	No	Yes	No	Yes
Shame	No	–	–	–		–	–
	Yes	–	–	–		–	–
Fear	No	254 (73.2)	23 (6.6)	–		–	–
	Yes	58 16.7)	12 (3.5)	–	-	–	–
Avoidance	No	261 (75.2)	24 (6.9)	260 (74.9)	25 (7.2)	–	–
	Yes	51 (14.7)	11 (3.2)	17 (4.9)	45 (13.0)	–	–
Discomfort	No	185 (53.3)	9 (2.6)	184 (53.0)	10 (2.9)	191 (55.0)	3 (0.9)
	Yes	127 (36.6)	26 (7.5)	93 (26.8)	60 (17.3)	94 (37.1)	59 (17.0)

Note: N = 347.

Table S2. Distribution of all the combinations of attitudes to dementia (persons and %).

**Shame**	**Avoidance**	**Fear**	**Discomfort = No**	**Discomfort = Yes**
No	No	No	175 (50.4%)	64 (18.4%)
		Yes	8 (2.3%)	14 (4.0%)
No	Yes	No	0	15 (4.3%)
		Yes	2 (0.6%)	34 (9.8%)
Yes	No	No	8 (2.3%)	13 (3.7%)
		Yes	0 (6.1%)	3 (0.9%)
Yes	Yes	No	1 (0.3%)	0
		Yes	0	10 (2.9%)

Note: N = 347.
